# The Effect of Dentin Exposure to Various Gutta‐Percha Solvents on the Bond Strength of Calcium‐Enriched Mixture Cement

**DOI:** 10.1155/ijod/3531603

**Published:** 2026-08-21

**Authors:** Alireza Adl, Fereshte Sobhnamayan, Mahdi Sedigh-shams, Yasaman Abadian

**Affiliations:** ^1^ Biomaterials Research Center, Department of Endodontics, School of Dentistry, Shiraz University of Medical Sciences, Shiraz, Iran, sums.ac.ir; ^2^ Department of Endodontics, Shiraz University of Medical Sciences, Shiraz, Iran, sums.ac.ir; ^3^ Student Research Committee, Shiraz University of Medical Sciences, Shiraz, Iran, sums.ac.ir

**Keywords:** calcium–enriched mixture cement, chloroform, dentin, eucalyptol, orange oil, push-out bond strength

## Abstract

**Introduction:**

The present study aimed to evaluate the bond strength between calcium‐enriched mixture (CEM) cement and dentin exposed to various gutta‐percha solvents.

**Method:**

A total of 105 dentin discs were obtained from the mid‐root of single‐rooted human teeth. The lumens were instrumented to achieve a standard diameter of 1.3 mm. The specimens were divided into a control group, which was not exposed to any solvents, and six experimental groups based on the solvents used (chloroform, eucalyptol, and orange oil) and exposure time (2 or 5 min). CEM cement was mixed and introduced into the lumens. The push‐out test was performed after 4 days. Statistical analyses were conducted using a one‐way ANOVA test (*p* = 0.05).

**Results:**

There were no statistically significant differences among the seven groups (*p* = 0.087).

**Conclusion:**

This study’s results show that the use of gutta‐percha solvents does not significantly affect the bond strength of CEM cement.

## 1. Introduction

Nonsurgical root canal retreatment is often indicated when the initial treatment has failed. Retreatment aims to remove the old filling material, effectively clean and shape the root canal system, and refill it [1].

Hand and rotary instruments are usually used alongside solvents to remove gutta‐percha, the most common root canal filling material [2, 3]. Although chloroform is the most commonly used and effective gutta‐percha solvent, its potential carcinogenicity has prompted the suggestion of alternative solvents such as eucalyptol, orange oil, and turpentine oil [4–6].

When solvents are used during endodontic retreatment, exposure of radicular and coronal dentin to them is inevitable. As these agents are strong lipid solvents, this exposure may alter the chemical composition and surface topography of dentin [7, 8], which, in turn, affects the adhesion of various materials to dentin [9, 10].

Calcium silicate cements (CSCs) have different applications in endodontic treatments, including vital pulp therapy, perforation repair, apical plug formation, and even root canal filling [11]. Mineral trioxide aggregate (MTA) was the first CSC to be introduced. However, due to its prolonged setting time, potential for discoloration, poor handling characteristics, and high cost [12, 13], various alternative materials have been introduced. Calcium‐enriched mixture (CEM) cement has the same clinical application as MTA but has a different chemical composition [14]. This cement has shown excellent biocompatibility, sealing ability, and hard tissue induction properties [15–18].

A recent study showed that when dentin was exposed to various gutta‐percha solvents, the bond strength of biodentine and capsule‐form MTA (CMTA) decreased, whereas white MTA (WMTA) was unaffected [9]. The bond strength of CEM cement has been investigated in various studies in the presence of irrigating solutions such as sodium hypochlorite and chlorhexidine [19, 20] as well as under blood contamination [21] and varying alkaline environments [22]. However, to our knowledge, there is no study on the bond strength of this cement to dentin treated by gutta‐percha solvents. In this regard, this study aimed to evaluate the effects of 2‐ and 5‐min exposures of root dentin to chloroform, eucalyptol, and orange oil on the bond strength of CEM cement. The null hypothesis (*H*_0_) was that exposure of root dentin to different gutta‐percha solvents (chloroform, eucalyptol, and orange oil) for 2 or 5 min would have no significant effect on the push‐out bond strength of CEM cement.

## 2. Materials and Method

Ethical approval for the study protocol was obtained from the Ethical Committee of the School of Dentistry, Shiraz University of Medical Sciences (Approval ID: IR.SUMS.REC.1397.123).

In this in vitro study, 60 freshly extracted, straight, single‐rooted human mandibular premolar teeth were selected and stored in 0.5% chloramine‐T until use (about 1 month) [9].

Teeth with root curvature, open apices, calcified canals, previous endodontic treatment, root resorption, or visible cracks were excluded from the study. Preoperative mesiodistal and buccolingual radiographs were taken to verify the presence of a single canal. The mid‐root of each tooth was sectioned using a cutting machine (Mecatome T180, PressiSA; Eybens, France) under a water spray to prepare two dentin discs, ~1.5 ± 0.2 mm thick, from each root. The lumina of dentin discs were enlarged using Gates Glidden drills (Dentsply, Switzerland) in sizes #2 to 5 to obtain a standardized diameter of 1.3 mm. All discs were examined under a stereomicroscope, and samples with cracks or oval lumina were excluded from the study. Finally, 105 dentin discs were selected and randomly allocated to the study groups *n* = 15 per group) in such a way that the two discs obtained from the same tooth were assigned to different groups. To remove the smear layer, discs were immersed in 17% ethylene diamine tetra‐acetic acid (EDTA) and 2.5% sodium hypochlorite (NaOCl) each for 3 min [9]. The samples were then immediately washed with distilled water and dried.

### 2.1. Sample Size Determination

The sample size was estimated using 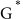
Power software (version 3.1.9.7, Universität Düsseldorf, Germany). The expected effect size was estimated from the magnitude of differences in push‐out bond strength reported by Sahebi et al. An alpha level of 0.05 and a statistical power of 80% were used for the sample size calculation. To compensate for possible specimen loss during specimen preparation and testing, the sample size was increased to 15 specimens per group.

Group 1: Not exposed to any solvent (Control).

Group 2: Exposed to eucalyptol (Consolidated‐Chemical; USA) for 2 min.

Group 3: Exposed to eucalyptol for 5 min.

Group 4: Exposed to orange oil (Morning pep, New York; USA) for 2 min.

Group 5: Exposed to orange oil for 5 min.

Group 6: Exposed to chloroform (Merck KGaA, Darmstadt, Germany) for 2 min.

Group 7: Exposed to chloroform for 5 min.

### 2.2. Sample Preparation

For solvent exposure, 0.2 mL of each solvent was placed into the disc lumens for 2 or 5 min.

The samples were then immediately washed in distilled water and dried with a gentle air spray for 5 s. CEM cement (BioniqueDent, Tehran, Iran) was prepared according to the manufacturer’s recommendations. The lumens of all samples were filled with CEM cement using an amalgam carrier and condensed with a hand plugger. A small, wet cotton pellet was used to remove excess material from the discs’ surfaces. The specimens were then wrapped in gauze soaked in normal saline and placed in sealed plastic containers. The specimens were incubated at 37°C for 3 days.

### 2.3. Push‐Out Testing

**Figure 1:**
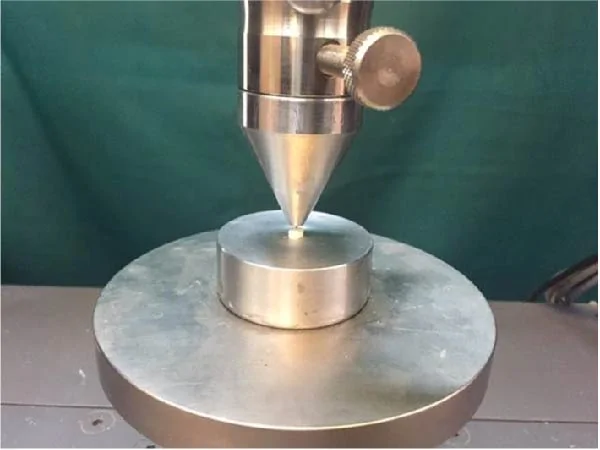
The push‐out bond strengths were measured using a universal testing machine (Z050, Zwick GmbH, Ulm, Germany).

The push‐out bond strengths were measured using a universal testing machine (Z050; Zwick/Roell, Ulm, Germany). The dentin discs were placed on a metal slab with a central hole to allow for the free motion of the plunger. The specimens were loaded with a 1‐mm‐diameter cylindrical stainless steel plunger at a speed of 1 mm/min (Figure 1). The maximum load applied to the CEM cement before dislodgement was recorded in Newtons (N). The bond strengths in MPa were expressed by dividing the recorded values (in N) by the adhesion surface area of the CEM cement (in mm^2^). The result was calculated according to the formula 2*πr* × *h*, where *π* is the constant (3.14), *r* is the root canal radius (0.65 mm), and *h* is the thickness of the root slice in mm [19]. The thickness of each dentin disc was measured using a digital caliper with an accuracy of 0.01 mm. The operator performing the push‐out test was blinded to the group allocation of the specimens as the discs were coded by a second investigator who did not participate in the testing procedure. The codes were revealed only after all the data had been recorded.

**Figure 2:**
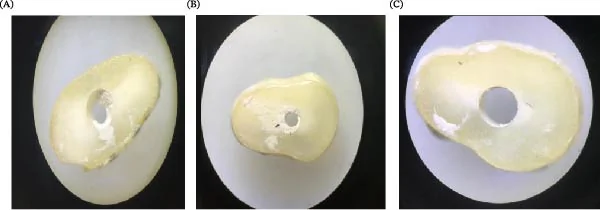
Different types of failure: (A) adhesive, (B) mix, (C) cohesive.

The slices were then examined using a stereomicroscope (BestScope, BS‐3060c, China) at 20 × magnification to determine the nature of the bond failure. As shown in Figure 2, each sample was categorized into one of three failure modes: adhesive failure at the CEM cement‐dentin interface, cohesive failure within the CEM cement, and mixed failure [19]. The normality of the bond strength data was assessed using the Shapiro–Wilk test. Because the data were skewed, a natural log transformation was applied prior to testing. After confirming normal distribution of the log‐transformed data (Shapiro–Wilk test, *p*  > 0.05). One‐way ANOVA was used to compare the bond strength values among the groups. The significance level was set at 0.05. Since the overall ANOVA result was not significant, no post‐hoc comparisons were performed.

## 3. Results

Three specimens were excluded due to breaking while being handled. Table 1 shows the mean ± standard deviation of bond strength values for all groups.

**Table 1:** Mean (±standard deviation) bond strength (MPa) of CEM cement exposed to different solvents for different durations.

Solvent	Time (min)	Number	Mean (SD)	95% CI	*p*	*η* ^2^
Control	—	15	7.26 ± (6.3)	3.7−10.7	0.087	0.11
Eucalyptol	2	15	4.91 ± (5.03)	2.1–7.7		
	5	15	6.19 ± (6.79)	2.4–9.9		
Orange oil	2	15	9.72 ± (5.35)	6.7−12.6		
	5	15	11.04 ± (6.76)	7.2−14.7		
Chloroform	2	15	6.66 ± (6.01)	3.3–9.9		
	5	12	7.51 ± (4.19)	4.8−10.1		

*Note:* No significant differences (*p*  > 0.05).

Abbreviations: *η* ^2^, partial eta squared effect size; *p*, *p*‐value; SD, standard deviation.

The highest and lowest bond strengths were reported in the orange oil group (5 min) and the eucalyptol group (2 min), respectively. However, there was no significant difference between the different groups (*p* = 0.087).

Table 2 shows the distribution of failure modes across the groups. Based on this table, no consistent pattern was observed among the experimental groups.

**Table 2:** Number (percentage) of different types of cement‐CEM bond failure in the presence of different solvents.

Solvent	Time (min)	Mix	Cohesive	Adhesive
Control	—	5 (33.33)	4 (26.66)	6 (40)
Eucalyptol	2	10 (66.66)	1 (6.66)	4 (26.66)
	5	6 (40)	0 (0)	9 (60)
Orange oil	2	7 (46.66)	6 (40)	2 (13.33)
	5	8 (53.33)	5 (33.33)	2 (13.33)
Chloroform	2	5 (33.33)	2 (13.33)	8 (53.33)
	5	4 (33.33)	0 (0)	8 (66.66)

## 4. Discussion

The present study evaluated the effect of exposure of dentin to the different gutta‐percha solvents on the bond strength of CEM cement. The proper bond strength of endodontic cements to dentin is a crucial physical property considered when evaluating their successful clinical use. A higher bond strength indicates close contact between the material and the dentinal wall, which determines a cement’s sealing ability and resistance to functional pressure and the mechanical forces of restorative material condensation [23]. Various techniques have been used to evaluate the bond strength between materials and dentin. Among them, the push‐out test has been reported to be the most efficient, reliable, and reproducible [22, 24]. This test is a standard method for measuring the interfacial shear strength between two surfaces and shows their resistance to dislodgement and adhesive properties [22].

Previous studies evaluating the bond strength of different materials to canal walls showed that the bond strength decreased in a coronal‐apical direction. Therefore, to eliminate the effect of the corono‐apical location of dentin on the results, we used only dentin discs obtained from the middle part of the roots. In the current study, the dentin discs were exposed to gutta‐percha solvents for 2 or 5 min, following similar studies [9, 10].

Like other CSCs, CEM cement can form hydroxyapatite and a hybrid layer between dentin and the cement [25]. Studies have noted that this hybrid layer formation and intratubular mineralization might improve the push‐out bond strength of biosilicate cements [25–28].

Gutta‐percha solvent may affect dentin structure, hybrid layer formation, and, consequently, the bond of CEM cement to dentin.

Studies evaluating the effect of gutta‐percha solvent on the physical properties of dentin have reported conflicting results. While a previous study showed that chloroform, xylene, and halothane significantly reduced the microhardness of enamel and dentin [8], another study reported that chloroform, eucalyptol, and orange oil did not decrease the microhardness of the root dentin [29]. Controversial findings have also been reported in studies evaluating the effects of solvents on the dentin mineral content. In one study, gutta‐percha solvents affected the mineral content of root dentin, particularly magnesium levels [30], whereas in another study, pure chloroform did not change dentin’s calcium or phosphorus levels [9].

The present study is the only study to evaluate the effect of different gutta‐percha solvents on the bond strength of CEM cement to dentin. Our findings show that none of the gutta‐percha solvents affected the push‐out bond strength of CEM cement to dentin after 2 or 5 min of exposure. Therefore, the null hypothesis (H0) that exposure to these solvents has no significant effect on the bond strength of CEM cement is not rejected. This finding is partially consistent with the study by Bayram et al. [9], in which none of the solvents tested (chloroform, orange oil, eucalyptol, and Endosolv) affected the bond strength of WMTA. However, the bond strength of Biodentine and CMTA to root dentin decreased. The researchers attributed these results to the longer setting time of WMTA in comparison with Biodentine and CMTA. Similarly, the results of the present study can be attributed to the setting time of CEM cement, which has been reported to be around 1 h [31]. Another possible explanation is the adhesion mechanism of the CEM cement. This calcium silicate‐based cement forms a mineral infiltration zone via ion exchange and apatite precipitation, a process that may be less dependent on the intact organic matrix of dentin, thereby making it less susceptible to solvent‐induced alterations. This interpretation aligns with the work of Ab Aziz et al. [32], who showed that exposure to gutta‐percha solvents for 5 min did not significantly affect the push‐out bond strength of iRoot SP, a premixed bioceramic sealer, to root dentin. Furthermore, a recent study by Mahjourianqomi et al. [33] demonstrated that the application of chloroform during retreatment was associated with significantly higher bond strength of EndoSeal MTA, another calcium silicate‐based sealer. These findings collectively suggest that calcium silicate‐based materials are not adversely affected by solvent‐induced changes in dentin, and, in some cases, bond strength may even increase following solvent exposure. From a clinical perspective, the present findings suggest that short‐term exposure of root dentin to chloroform, eucalyptol, or orange oil did not adversely affect the immediate push‐out bond strength of CEM cement under the experimental conditions of this study. This observation may be relevant in clinical situations where CEM cement is placed following retreatment procedures, such as perforation repair, apical barrier formation, or regenerative endodontic procedures. However, these results should be interpreted with caution as the in vitro design and controlled laboratory conditions may not fully reflect clinical performance. Moreover, the findings are limited to the immediate bond strength after solvent exposure and thorough washing and do not account for the potential effects of residual solvent traces, dissolved gutta‐percha remnants, or long‐term intraoral aging. Therefore, further in vivo and long‐term studies are required before definitive clinical recommendations can be made.

It should be noted that although no statistically significant differences were observed among the groups, the highest bond strength values were observed in the orange oil groups. This trend may indicate a potentially favorable effect of this solvent on the dentin surface characteristics or CEM cement adhesion. However, due to the absence of statistical significance and considerable variability among samples, this finding should be interpreted with caution and cannot be considered conclusive.

In the present study, no consistent pattern was observed in the distribution of failure modes among the experimental groups (Table 2). Previous studies evaluating the push‐out bond strength of CEM cement have also reported contradictory findings, with some demonstrating predominantly cohesive failures [19, 20, 34], while others have reported mainly adhesive [35] or mixed [36, 37] failures. This inconsistency may be attributed to variations in dentin substrate conditions, canal preparation protocols, irrigant type and exposure time, cement handling and placement technique, and differences in specimen sectioning and testing methodologies.

In general, a cohesive failure indicates stronger adhesion to dentin than the internal strength of the cement itself, whereas adhesive failure reflects a weaker bond at the dentin–cement interface. In the present study, the orange oil groups showed a higher incidence of cohesive and mixed failures, suggesting acceptable interaction between orange oil‐treated dentin and CEM cement (Table 2). However, because no statistically significant differences were detected in bond strength values and considerable variability was present, this observation should be interpreted cautiously and cannot be considered conclusive (Table 1).

The present study has several limitations that should be considered when interpreting the findings. First, this was an in vitro investigation performed under controlled laboratory conditions, which may not fully reproduce the biological and mechanical complexities of the clinical environment. Second, the push‐out bond strength was evaluated only 4 days after placement of the CEM cement, and no artificial aging or thermocycling was performed. Therefore, the long‐term durability of the dentin–cement interface after solvent exposure remains unclear. Third, standardized dentin discs with uniform lumen diameters were used to minimize experimental variability; however, this model does not fully replicate the anatomical complexity, irregularities, and dimensional variations of natural root canal systems encountered clinically. Moreover, another limitation of the present study is that two dentin discs were obtained from each tooth. Although the two discs from the same tooth were always allocated to different experimental groups to avoid within‐group pseudoreplication, some degree of biological dependence between specimens originating from the same tooth cannot be completely excluded. Therefore, the findings should be interpreted with this consideration in mind.

Finally, during clinical retreatment procedures, gutta‐percha solvents are applied in the presence of remnants of the filling material, and dentin may be exposed to residual dissolved gutta‐percha and traces of the solvent. These conditions were not simulated in the present study, which evaluated solvent‐exposed dentin after thorough washing. Future studies should incorporate aging simulations and more clinically relevant retreatment conditions to clarify better the long‐term effect of gutta‐percha solvents on the bond strength of CEM cement.

## Author Contributions

Conceptualization and study validation: Alireza Adl and Fereshte Sobhnamayan. Implementation and supervision: Alireza Adl, Fereshte Sobhnamayan, and Mahdi Sedigh‐shams. Writing and reviewing: Alireza Adl, Mahdi Sedigh‐shams, and Yasaman Abadian. Funding acquisition: Fereshte Sobhnamayan and Yasaman Abadian.

## Funding

The current study received funding from the Vice‐Chancellery of Shiraz University of Medical Sciences (Grant 2007).

## Conflicts of Interest

The authors declare no conflicts of interest.

## Data Availability

The data that support the findings of this study are available from the corresponding author upon reasonable request.
